# Genome-Wide Identification and Expression Analysis of 1-Aminocyclopropane-1-Carboxylate Synthase (ACS) Gene Family in *Myrica rubra*

**DOI:** 10.3390/ijms26104580

**Published:** 2025-05-10

**Authors:** Huanhui Huang, Xintong Liu, Yiqing Liu, Fangli Wu, Weibo Jin

**Affiliations:** 1 Key Laboratory of Plant Secondary Metabolism and Regulation of Zhejiang Province, College of Life Science and Medicine, Zhejiang Sci-Tech University, Hangzhou 310018, China; hhh00081919@163.com (H.H.); 2023220902026@mails.zstu.edu.cn (X.L.); 2023210901042@mails.zstu.edu.cn (Y.L.); wfl@zstu.edu.cn (F.W.); 2 Shaoxing Academy of Biomedicine, Zhejiang Sci-Tech University, Shaoxing 312366, China

**Keywords:** *Myrica rubra*, ACS gene, genome-wide, expression patterns

## Abstract

Ethylene plays a crucial role in plant growth, development, and stress responses, with 1-aminocyclopropane-1-carboxylate synthase (ACS) being a key enzyme in its biosynthetic pathway. However, the ACS gene family of *Myrica rubra* has not yet been systematically identified and characterized. In this study, we identified and characterized seven ACS genes (*MrACS*) in *Myrica rubra* through genome-wide analysis. Phylogenetic analysis revealed that these genes belong to three major subfamilies, with certain members clustering closely with ACS genes from Rosaceae species, suggesting a conserved evolutionary relationship. Gene structure and the conserved motif analyses confirmed functional conservation, while chromosomal localization indicated an uneven distribution across the genome. Collinearity analysis revealed strong homologous relationships between *Myrica rubra* and other plant species, particularly *Solanum lycopersicum*, *Vitis vinifera*, and *Prunus persica*. Furthermore, the transcriptome data demonstrated distinct temporal and tissue-specific expression patterns, with *MrACS5* showing fruit-specific expression, suggesting its potential role in fruit ripening. These findings provide comprehensive insights into the ACS gene family in *Myrica rubra*, offering a valuable foundation for further functional studies on ethylene biosynthesis and its regulatory mechanisms in fruit development.

## 1. Introduction

*Myrica rubra*, commonly known as Chinese bayberry, is a subtropical evergreen tree in the Myricaceae family that is native to southern China. Its fruit, prized for its distinctive sweet-and-sour flavor and high nutrimental value, is widely favored by consumers and makes a substantial contribution to the local economy [1]. Beyond its commercial appeal, *Myrica rubra* has attracted increasing scientific attention due to its potential health-promoting properties [2]. Previous studies have demonstrated that its fruit contains abundant bioactive compounds, such as anthocyanins, ascorbic acid, phenolic acids, and flavonols, which exhibit strong antioxidant, anti-inflammatory, antibacterial, and antitumor activities. These bioactive constituents are believed to contribute to the therapeutic potential of *Myrica rubra*, making it a promising candidate for further pharmacological and nutraceutical investigations [3,4,5]. The ripening period of *Myrica rubra* generally ranges from late May to early July, varying according to cultivar and geographical location. Due to its relatively short maturation window, optimal flavor is achieved only when the fruit is harvested at full ripeness. However, improper postharvest handling often leads to rapid softening and decay, resulting in substantial economic losses. Therefore, understanding the molecular mechanisms underlying fruit ripening in *Myrica rubra* is crucial for both improving postharvest management strategies and enhancing the commercial value of this species.

Fruit ripening is a complex physiological process regulated by diverse internal and external factors, including temperature, light, nutrients, hormones, epigenetic regulators, and endogenous signaling pathways [6,7]. Among these factors, ethylene is the best-characterized and most direct regulator of the fruit ripening process [8]. Ethylene, a gaseous plant hormone, governs a wide range of developmental processes, such as seed germination, fruit maturation, organ senescence, and stress responses [9,10,11]. The ethylene biosynthesis pathway is relatively simple, involving two key enzymatic steps. First, S-adenosyl-L-methionine (SAM) is converted into 1-aminocyclopropane-1-carboxylic acid (ACC) by ACC synthase (ACS), which catalyzes the rate-limiting step and serves as a key regulatory point in ethylene production [12,13]. Next, ACC is converted into ethylene by ACC oxidase (ACO). ACS genes play pivotal roles in ripening regulation across diverse species. For instance, *PpACS1a* is crucial for pear ripening, showing increased expression during the ripening stage [14]. *ACS1* plays a dominant role in blueberry ripening, with cultivar-specific expression patterns [15]. In octoploid strawberry plants, *FaACS27* and *FaACS29* are highly expressed during achene development and contribute to ripening regulation [16]. In apples, *MdACS3a* regulates ripening, while *MdACS6* acts at earlier developmental stages [17,18]. In tomatoes, RNA interference (RNAi) targeting *ACS6*, *ACS1*, and *ACS2* suppresses ethylene biosynthesis, delays ripening, and extends shelf life [19]. In bananas, a multilayered regulatory cascade involving *MaXB3*, *MaNAC*, and *MaERF11* modulates ripening, wherein *MaXB3* negatively regulates ethylene biosynthesis by promoting the degradation of *MaNAC2*, *MaACS1*, and *MaACO1* [20].

Beyond fruit ripening, ACS genes play essential roles in various aspects of plant growth and development by regulating ethylene biosynthesis. These roles include seed germination, root and shoot elongation, floral organ development, fruit set, leaf senescence, and organ abscission [21,22]. In *Arabidopsis*, *AtACS7* is highly expressed during seed germination and contributes to early seedling development [23]. Mutations in *AtACS4* and *AtACS8* reduce ethylene production and affect lateral root formation [24]. *AtACS2* and *AtACS6* participate in petal and stamen development, with mutations resulting in floral defects or infertility [25]. In tomato plants, *SlACS2* plays a critical role in floral organ development and normal fruit set, while *SlACS2* and *SlACS4* are key regulators of fruit softening and color change during ripening [26]. In the Cucurbitaceae, ACS genes are involved in sex determination. For example, *CmACS6*, *CmACS7*, *CmACS9*, and *CmACS11* in melon, and *ClACS1*, *ClACS7*, and *ClACS11* in watermelon, are predominantly expressed in female flowers, while *ClACS6* shows high levels of expression in male flowers [27]. In Chinese chestnut (*Castanea mollissima*), *CmACS7* expression determines ovule fertility, and its overexpression prior to fertilization leads to excessive ACC accumulation and ovule abortion [28]. Moreover, a natural allelic variant of *ACS7* in watermelon plants modulates primary root elongation through ethylene-mediated signaling [29]. Transcriptional regulation of the ACS genes by various transcription factors has also been reported. In Arabidopsis, *WRKY22* promotes ethylene biosynthesis by activating ACS5 and ACO5, thereby modulating root development [30], while *WRKY71* regulates ethylene-mediated leaf senescence by activating *EIN2*, *ORE1*, and *ACS2* [31].

Increasing research evidence shows that ACS genes are responsive to both biotic and abiotic stresses, thereby modulating ethylene production to enhance stress adaptation [32,33]. In rice, *OsACS5* is strongly upregulated under hypoxic stress, promoting adventitious root formation through ethylene accumulation [34]. In bananas, *MaACS1* and *MaACS14* display distinct expression patterns under low-potassium and low-nitrogen stress [35]. Elsewhere, the ethylene response factor *GsERF1* improves aluminum tolerance in Arabidopsis by upregulating *ACS4*, *ACS5*, and *ACS6* [36]. Similarly, glutathione enhances resistance to necrotrophic pathogens and salt stress by triggering the *WRKY33*-mediated activation of *ACS2* and *ACS6* [37]. In cotton plants, the expression patterns of *GhACS10* and *GhACS12* change under various abiotic stresses, including cold, heat, drought, and salinity [38]. In sugarcane, *ACS2* and *ACS3* respond to low-nitrogen stress by regulating ethylene biosynthesis, contributing to stress tolerance and sugar accumulation [39]. These findings highlight the central role of ACS genes in regulating ethylene signaling in response to environmental stimuli.

Given the importance of the ACS genes in ethylene biosynthesis and their diverse roles in plant development and stress adaptation, investigating their structure, evolution, and expression patterns in various plants is essential. *Myrica rubra* genome sequencing was recently completed and published. However, the ACS gene family of this species has still not been reported. For this study, we performed a genome-wide identification of the ACS gene family in *Myrica rubra* and investigated their phylogenetic relationships, gene structures, conserved motifs, and chromosomal distributions. In addition, synteny analysis was conducted to evaluate evolutionary conservation, and gene expression profiles were analyzed to explore their potential roles during fruit development. These findings lay a solid foundation for future studies on ethylene biosynthesis and fruit ripening in *Myrica rubra* and related species.

## 2. Results

### 2.1. Identification of the ACS Gene Family in Myrica rubra

**Figure 1:**
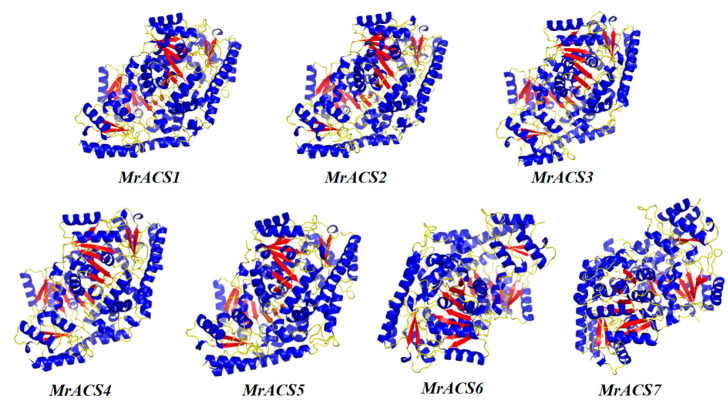
Predicted tertiary structures of seven MrACS proteins. Blue regions represent α-helices, red regions indicate β-sheets, and yellow regions denote random coils.

A total of seven *MrACS* gene family members in *Myrica rubra* were identified using a combination of BLAST (version 2.16.0) and HMMER (version 3.4) searches (Table 1 and Table S1). These proteins range in length from 446 amino acids (*MrACS3*) to 551 amino acids (*MrACS7*), with molecular weights (MW) of between 50.18 kDa and 60.34 kDa. The isoelectric points (pI) vary from 5.84 (*MrACS2*) to 8.33 (*MrACS7*), suggesting differences in protein charge. The aliphatic index for these proteins ranged from 82.6 to 89.6, and the grand average of hydropathicity (GRAVY) values was consistently negative, indicating that the *MrACS* proteins are hydrophilic. Subcellular localization predictions revealed that five of the *MrACS* proteins were localized in the cytoplasm, while two were localized in the nucleus. Additionally, the predicted tertiary structures showed that all *MrACS* proteins are composed of abundant α-helices and β-sheets, exhibiting overall compact three-dimensional structures (Figure 1).

### 2.2. Phylogenetic Analysis of the MrACS Gene Family

**Figure 2:**
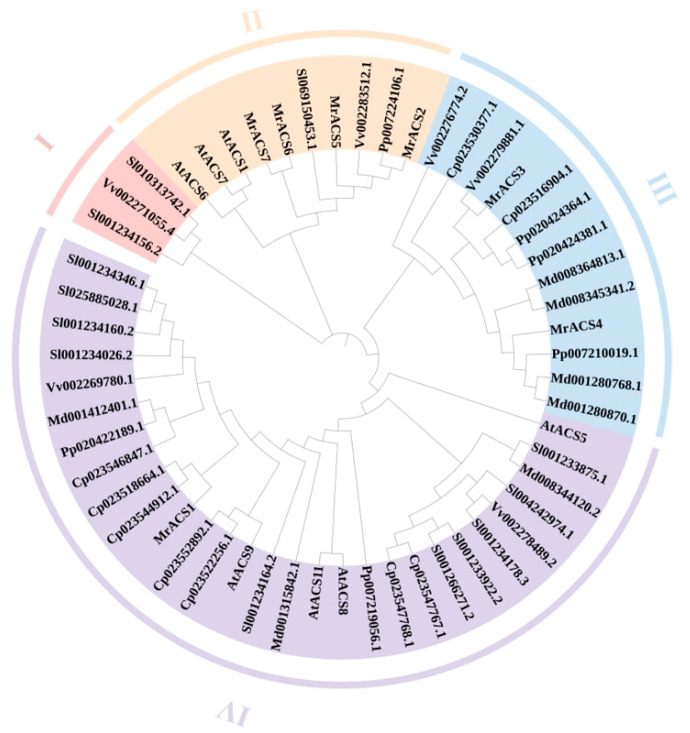
Phylogenetic relationships of the ACS proteins in *Myrica rubra* and six other species. This neighbor-joining phylogenetic tree was constructed based on a multiple sequence alignment of 55 ACS protein sequences from seven species. Different colors represent different subfamilies (I–IV).

To access the evolutionary relationships among ACS genes, a phylogenetic tree was constructed based on the full-length protein sequences from seven plant species, including *Myrica rubra* (7 *MrACS*s), *Arabidopsis thaliana* (7 *AtACS*s), *Malus domestica* (7 *MdACS*s), *Prunus persica* (6 *PpACS*s), *Solanum lycopersicum* (13 *SlACS*s), *Cucurbita pepo* (9 *CpACS*s), and *Vitis vinifera* (6 *VvACS*s). A total of 55 ACS protein sequences were aligned and analyzed using MEGA 7.0 software (Supplementary Table S2). The resulting phylogenetic tree revealed that the ACS gene family could be classified into three major clades. The largest clade comprised 42 members, which were further subdivided into two main subgroups (Clade III and Clade IV). Clade I and Clade II contained 3 and 10 members, respectively, each also forming two subgroups (Figure 2). *MrACS* genes were distributed across different clades, with several clustering closely with homologs from the Rosaceae species such as peach and apple, suggesting a shared ancestry or conserved selection pressure.

### 2.3. Gene Structure and Conserved Motif Analysis of MrACSs

**Figure 3:**
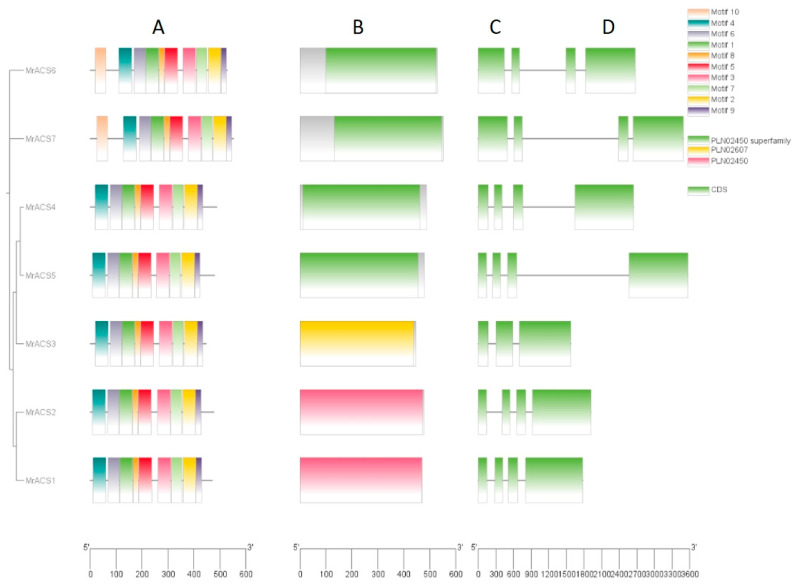
Phylogenetic analysis, gene structure, conserved domains, and conserved motifs of the *MrACS* gene family. (**A**) Phylogenetic tree of the *MrACS* proteins. (**B**) Conserved motif distribution of the *MrACS* proteins. Motifs 1–10 are shown as colored boxes, with different colors representing distinct motifs. (**C**) Conserved domain architecture of the *MrACS* proteins. (**D**) Exon-intron structures of the *MrACS* genes. Green boxes represent exons, and black lines represent introns.

To further investigate the structural characteristics and evolutionary relationships of the *MrACS* gene family, conserved domains, protein motifs, and gene structures were analyzed using NCBI Batch CD-search (Bethesda, Maryland, USA), MEME (Knoxville, Tennessee, USA), and TBtools (Guangzhou, China). All *MrACS* proteins, except for *MrACS3*, contained the conserved PLN02450 domain (Figure 3), which is characteristic of the ACS protein family. The gene structure analysis revealed that *MrACS* genes contain between three and four exons, indicating relatively conserved exon-intron organization. Ten conserved motifs were identified among the *MrACS* proteins. Notably, *MrACS1* and *MrACS2* were each missing one motif, whereas the remaining members shared nine common motifs (Supplementary Table S3). These findings, in conjunction with the identified phylogenetic relationships, suggested that closely related members within the same clade exhibit highly similar motif compositions. This pattern supports the hypothesis that ACS subfamilies may possess conserved functional roles.

### 2.4. Chromosomal Localization and Collinearity Analysis of the MrACS Genes

**Figure 4:**
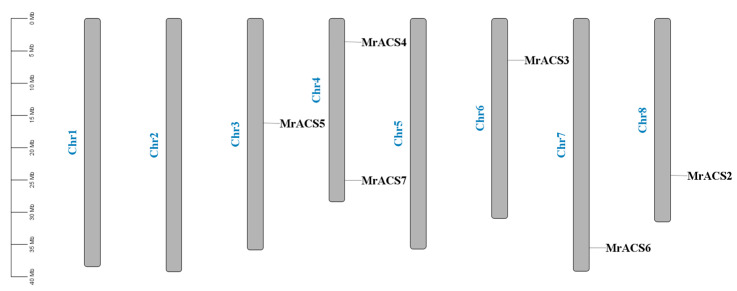
Schematic representations for the chromosomal distribution of *MrACS* genes.

**Figure 5:**
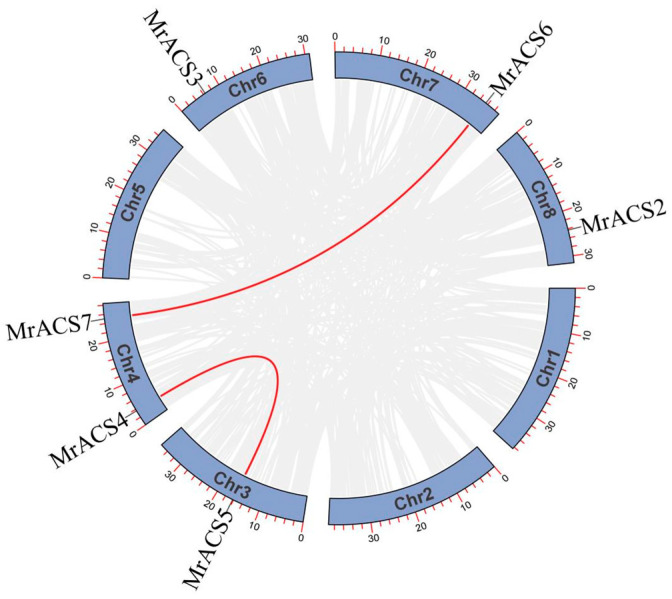
Distribution and duplication events of *MrACS* genes across the bayberry genome. The gray lines indicate synteny blocks in the poplar genome, and the red lines indicate the synteny gene pairs of the ACS gene family.

The results of the chromosomal localization analysis showed that the seven *MrACS* genes were distributed on chromosomes 3, 4, 6, 7, and 8, with *MrACS4* and *MrACS7* both located on chromosome 4, while *MrACS1* was located on an unanchored scaffold (Figure 4). In addition, the collinearity analysis revealed that two gene duplication events were identified: *MrACS4*–*MrACS5* and *MrACS6*–*MrACS7* (Figure 5). Ka/Ks ratio analysis showed values of 0.09 and 0.16 for these gene pairs, respectively (Table 2), indicating that the purifying selection process has acted to conserve their functions.

**Figure 6:**
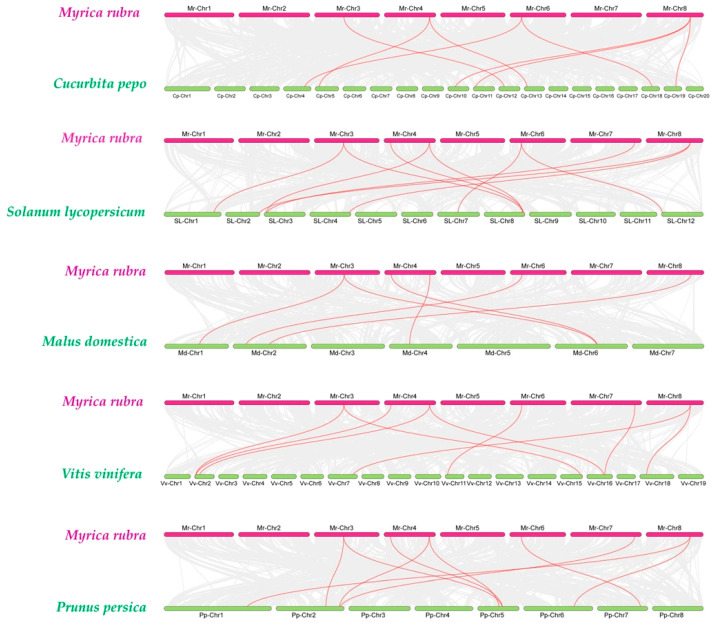
Synteny analysis of ACS genes between *Myrica rubra* and *Cucurbita pepo*, *Solanum lycopersicum*, *Malus domestica*, *Vitis vinifera*, and *Prunus persica*. Gray lines in the background indicate the collinear blocks within *Myrica rubra* and other plant genomes, and the red lines indicate the ACS gene pairs. The chromosomes of *Myrica rubra* are labeled in magenta, while chromosomes of the other species are labeled in green.

To further explore the evolutionary conservation and divergence of the *MrACS* gene family, synteny analyses were conducted with three fruit crops (comprising *Malus domestica*, *Vitis vinifera*, and *Prunus persica*) and two vegetable crops (comprising *Cucurbita pepo* and *Solanum lycopersicum*). The results revealed 10 homologous gene pairs between bayberry and tomato, 9 pairs with grape and peach, and 6 and 8 pairs with apple and pumpkin, respectively (Figure 6). These syntenic relationships suggest that the ACS genes in these species may share a common ancestral origin and that gene duplication or conservation events likely occurred after the divergence of these plant lineages.

### 2.5. Expression Patterns of MrACS Genes in Myrica rubra

**Figure 7:**
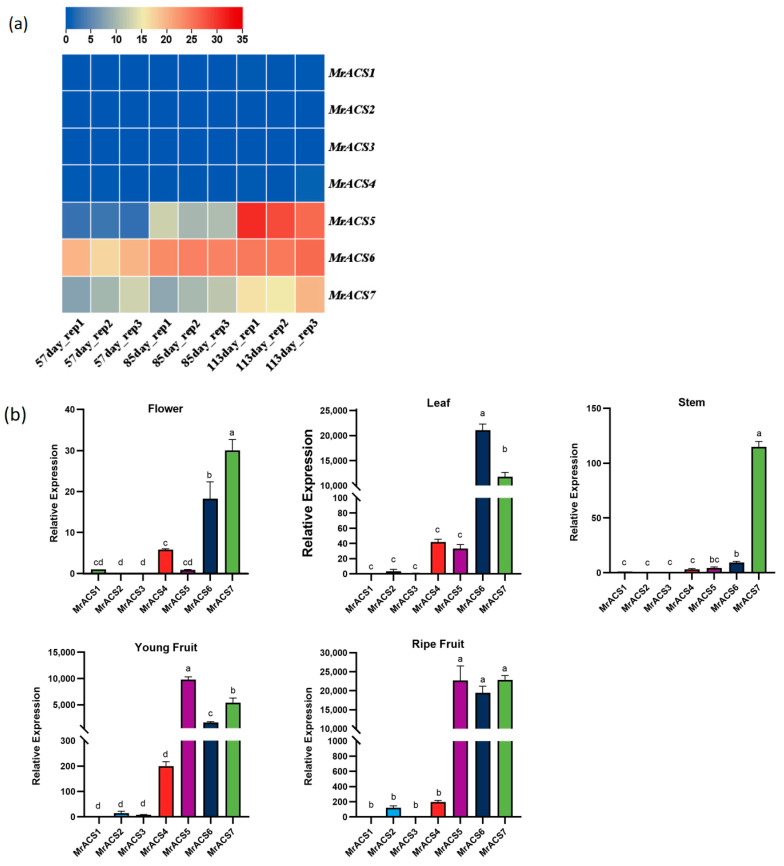
Expression patterns of *MrACS* genes in bayberry after pollination. (**a**) The heatmap shows the expression levels of *MrACS* genes on the 57th day, 85th day, and 113th day after pollination. (**b**) Relative expression levels of *MrACS1-7* in the flower, leaf, stem, immature fruit, and ripe fruit. The results are expressed as the means ± SEM of three biological replicates. Different letters indicate extremely significant differences at *p* < 0.01, according to a one-way analysis of variance (ANOVA) with Tukey’s multiple comparisons test.

To investigate the expression profiles of *MrACS* genes, transcripts per million (TPM) values were calculated (Table S4), based on the RNA-seq data in *Myrica rubra,* at three time points of 57, 85, and 113 days after pollination (DAP). The expression levels of three genes (*MrACS5*, *MrACS6*, and *MrACS7*) increased with the increase in pollination days, while the expression levels of the other four *MrACS* genes remained unchanged (Figure 7a). To further explore the tissue-specific expression of the *MrACS* genes, the expression levels in *Myrica rubra* in the flowers, leaves, stems, and fruits were examined (Figure 7b). *MrACS1*, *MrACS2*, and *MrACS3* exhibited consistently low expression across all tissues, indicating limited or possibly redundant roles in *Myrica rubra*. In contrast, the remaining four genes showed variable and tissue-specific expression patterns. *MrACS7* was highly expressed in all tissues, with higher levels than *MrACS6*, implying a more ubiquitous regulatory function. *MrACS4* showed low expression in flowers, whereas *MrACS5* was specifically and highly expressed in fruit, with minimal expression in other tissues. These expression profiles suggest that *MrACS* genes may be functionally diversified and may play distinct roles during fruit development and in different tissues of *Myrica rubra*.

## 3. Discussion

Ethylene is a key regulator in plant growth, development, fruit ripening, and stress tolerance [9,40]. The enzyme 1-aminocyclopropane-1-carboxylate synthase (ACS) catalyzes the rate-limiting step in ethylene biosynthesis and is encoded by a multigene family [13]. In *Myrica rubra*, we identified seven ACS genes, providing insight into the genetic basis of ethylene production in this economically and ecologically important species. Gene structure analysis revealed conserved exon-intron organization among the *MrACS* genes, which is consistent with observations in other species such as *Malus domestica* and *Prunus persica* [17,41]. Chromosomal localization analysis showed that the *MrACS* genes are unevenly distributed across chromosomes, a pattern that is frequently observed in plant genomes and is often associated with segmental duplications or genome rearrangements [42]. In this study, the gene pairs *MrACS4–MrACS5* and *MrACS6–MrACS7* exhibited low Ka/Ks ratios, indicating that the purifying selection process had maintained their functional integrity. This suggests that these duplicated genes are under evolutionary constraints that preserve their roles in ethylene biosynthesis. Phylogenetic analysis confirmed that these *MrACS* genes are highly conserved and are closely related to ACS genes from members of the Rosaceae family, implying shared evolutionary pressures, particularly those related to fruit development and ripening [43]. Collinearity analysis also provided compelling evidence of conserved homologous relationships between *Myrica rubra* and other plant species, particularly *Vitis vinifera* and *Prunus persica*, highlighting the functional significance of ACS genes in ethylene-mediated physiological processes across diverse fruit-bearing species. The distribution of ACS genes across multiple chromosomes in *Myrica rubra* suggests that gene duplication events, including both segmental and tandem duplications, have likely played a pivotal role in the expansion of this gene family. Such duplication events have been widely documented in other plant species, where they contribute to the functional diversification and adaptive evolution of ethylene biosynthesis pathways [44]. The uneven distribution of *MrACS* genes across the *Myrica rubra* chromosomes indicates that their genomic arrangement is independent of chromosome size. Similar patterns have been observed in *Arabidopsis thaliana* and *Oryza sativa*, where gene density and the chromosomal positioning of ACS genes are shaped by evolutionary constraints and regulatory interactions, rather than genome size alone [45]. Further comparative genomic analyses, especially those focusing on gene duplication mechanisms and the role of regulatory interactions, could offer deeper insights into the evolutionary forces shaping the distribution and functional diversification of the ACS gene family in *Myrica rubra*.

Tissue-specific expression patterns of ACS genes often reflect functional divergence following gene duplication. In *Myrica rubra*, RT-qPCR analysis revealed that *MrACS5* was predominantly expressed in fruit tissue, which is consistent with a specialized role in ripening-related ethylene production, similar to *SlACS2*/*4* in tomatoes and *MdACS1* in apples [46,47,48,49]. Conversely, *MrACS1*-*3* exhibited uniformly low expression levels across tissues, possibly indicating low basal activity or inducibility under specific conditions, as seen with *AtACS6* in *Arabidopsis thaliana* [50]. *MrACS7* showed ubiquitous expression across all tissues, implying a conserved housekeeping role in basal ethylene biosynthesis. *MrACS4* was moderately expressed in the vegetative organs and may be involved in processes like development or senescence. Comparable findings have been reported in Cucurbita maxima, where ethylene biosynthesis genes like *CmaACS4*, *CmaACS7*, and *CmaACS9* display flower-type-specific expression, reinforcing the notion of functional divergence within the gene family [33]. These distinct expression patterns underscore the regulatory specialization of *MrACS* genes, which is potentially governed by promoter architecture, epigenetic modifications, and tissue-specific transcription factor interactions. Further mechanistic studies are essential to elucidate how these regulatory networks shape the spatiotemporal roles of *MrACS* genes in *Myrica rubra*.

Fruit ripening is a genetically programmed and hormone-regulated process, with ethylene playing a central role in climacteric fruits such as tomatoes and apples [51,52,53]. In tomatoes, the transcription factor NAC-like1 can bind to the promoters of the ethylene biosynthesis genes *SlACS2* and *SlACS4*, thereby promoting ethylene production [47]. Additionally, in RIN- and NOR-deficient tomato mutants, the accumulation of ACS and ACO mRNAs is significantly suppressed, resulting in delayed fruit ripening [54,55]. In apples, *MdERF2* represses *MdACS1* transcription, and *MdACS3* mutations extend shelf life [17,56]. Compared with these model systems, the molecular regulation of fruit ripening in *Myrica rubra* remains largely unexplored. However, the present study identified *MrACS5* as a gene that is highly expressed during fruit development, suggesting its involvement in ethylene biosynthesis and fruit ripening. Unlike tomatoes or bananas, where multiple ACS genes are simultaneously activated during ripening, *MrACS5* exhibited tissue- and stage-specific expression, indicating a potentially distinct and more specialized regulatory mechanism. Notably, the weak and constitutive expression of other *MrACS* genes implies that they may function under specific environmental stimuli or hormonal cues, rather than in general developmental regulation.

Beyond plant development, ACS genes are known to mediate responses to biotic and abiotic stresses. Ethylene, synthesized via the ACS pathways, functions as a critical signaling molecule in stress adaptation [57,58]. In Arabidopsis thaliana, for example, *AtACS8* mediates Cu^2+^-induced ethylene production and contributes to pathogen defense [59], while *AtACS4/5/6* genes are upregulated by *GsERF1* under aluminum stress [36]. Pathogen-induced ethylene biosynthesis involves *AtACS2/6* activation via MPK3/6 phosphorylation, linking MAPK cascades to hormone regulation [60]. In rice, the *OsACS1/2* genes respond dynamically to flooding and hypoxia, supporting adaptive ethylene synthesis [61]. Ethylene also interacts with SA and JA signaling to fine-tune immune responses under biotic stress [62]. In kiwifruit, ASA suppresses ethylene biosynthesis by repressing *AdACS1/2* transcription and destabilizing *AdACS3* through the inhibition of AdMPK16-mediated phosphorylation, revealing multi-level regulation [63]. Although ACS genes are known to mediate stress responses in model plants, their roles in Myrica rubra remain unclear. Our results have revealed the tissue-specific expression of *MrACS* genes, but their response to stress has not yet been explored. Future studies should investigate their stress inducibility and potential involvement in ethylene-mediated adaptation. In addition, the possible link between ethylene biosynthesis and bioactive compound regulation in *Myrica rubra* merits further research.

## 4. Materials and Methods

### 4.1. Plant Materials

*Myrica rubra* was cultivated in an orchard located in Wenling City, Zhejiang Province, China. Trees with similar growth potential and identical environmental conditions were selected for the study. Samples were collected from all tissues of *Myrica rubra*, with at least three biological replicates per sample. All samples were immediately snap-frozen in liquid nitrogen and stored at −80 °C for subsequent analysis.

### 4.2. Identification of ACS Genes in Myrica rubra

The methodology followed in this study was based on the approach outlined by Xie et al. [64]. The genome sequence, assembly files, and annotation information of *Myrica rubra* were retrieved from the *Myrica rubra* database (http://www.bayberrybase.cn/ (accessed on 1 November 2024)) [65]. The Arabidopsis ACS gene and protein sequences were obtained from the Arabidopsis database (TAIR) (https://www.arabidopsis.org/ (accessed on 10 November 2024)) [66]. The hidden Markov models (HMMs) of the ACS gene family conserved domain (PF00155) were sourced from the Pfam protein family database (http://pfam-legacy.xfam.org/ (accessed on 1 December 2024)) [67]. To identify potential *Myrica rubra* ACS genes, HMMER (Cambridge, UK) and BLASTP (Bethesda, Maryland, USA) tools were employed, along with the ACS domain model and Arabidopsis ACS protein sequence retrieved from the Pfam database. First, the Arabidopsis ACS protein sequences were used as the query sequences for BLASTP screening on the entire protein sequence of *Myrica rubra* (E-value < 1 × 10^−5^ and identity > = 40). Initially, *Myrica rubra* ACS gene sequences were used as the query sequence for a reverse BLASTP alignment (E-value < 1 × 10^−10^). ACS proteins in the *Myrica rubra* genome were further identified using the HMM search program in TBtools. Redundant sequences were removed from the results. To verify the presence of the ACS conserved domain, candidate ACS genes were compared against the NCBI-CDD (https://www.ncbi.nlm.nih.gov/ (accessed on 15 December 2024)) database. The conserved domains of candidate ACS genes were compared to verify whether they contained ACS conserved domains, using the online program ExPASY (https://web.expasy.org/compute_pi/ (accessed on 20 December 2024)) [68]. Protein properties such as molecular weight (MW) and the isoelectric point (PI) of each ACS protein were calculated. The subcellular localization of the ACS protein in *Myrica rubra* was predicted using WoLF PSORT (https://wolfpsort.hgc.jp/ (accessed on 25 December 2024)).

### 4.3. Phylogenetic Analysis of ACS Genes in Myrica rubra

The ACS protein sequences of *Arabidopsis thaliana*, pumpkin (*Cucurbita pepo*), apple (*Malus domestica*), peach (*Prunus persica*), tomato (*Solanum lycopersicum*), grape (*Vitis vinifera*), and bayberry (*Myrica rubra*) were aligned multiple times using MEGA software, and a phylogenetic tree was constructed using the neighbor-joining (NJ) method.

### 4.4. Chromosome Distribution and Collinearity Analysis of ACS Genes in Myrica rubra

Genomic data for these species were downloaded from the NCBI website (https://www.ncbi.nlm.nih.gov/). The Btools-GXF Re-build tool was used to extract position annotations for all coding sequences (CDS) from the *Myrica rubra* gene assembly file. Based on these annotations, a chromosome coordinate file for the ACS gene family was generated, and the gene locations were visualized across the eight chromosomes using TBtools software, version 2.225. To assess the synteny relationships between *Myrica rubra* and these species, the TBtools Multiple Synteny Plot tool (Guangzhou, China) was employed, and the syntenic patterns were visualized. Multiple collinear scanning tool (MCScanX) was used to evaluate the interspecies replication events of bayberry.

### 4.5. Analysis of ACS Gene Structure and Conserved Motifs in Myrica rubra

The exon-intron structure of the ACS gene was visualized using TBtools. Conserved motif analysis was conducted using the MEME suite (https://meme-suite.org/meme/doc/meme.html) [69], with the motif number parameter set to 10. The conserved motif results identified in the bayberry ACS gene family members were visualized using TBtools software.

### 4.6. Expression Profiling of MrACS Genes via Transcriptome Analysis and Quantitative Real-Time PCR

Transcriptome data from 57, 85, and 113 days after pollination (DAP) were selected to represent key stages of *Myrica rubra* fruit development—namely, immature, color-break, and fully ripe stages—during which ethylene biosynthesis and sugar accumulation are actively modulated [70]. The associated RNA-seq accession numbers are provided in Table S5 and are publicly available via the NCBI.

Total RNA was extracted using the Fast Pure Universal Plant Total RNA Extraction Kit (Vazyme, Nanjing, China), according to the manufacturer’s protocol. The RNA purity and concentration were analyzed using Nanodrop2000, and RNA integrity was assessed by 2% agarose gel electrophoresis. cDNA was performed following the manufacturer’s instructions by Prime Script™RT Kit (one-step gDNA removal) (Takara, Shiga, Japan). Specific primers for qRT-PCR were designed using Primer Premier v.5.0. SYBR Green PCR was carried out using a qTOWER3G fluorescence quantitative PCR instrument (Jena, Germany). In each reaction, 1 µL of the cDNA template, 10 µL of Taq Pro universal SYBR qPCR Master Mix (Vazyme, Nanjing, China), and 0.2 µm of specific primers were mixed, and ddH_2_O was added to a volume of 20 µL. The reaction was pre-denatured at 95 °C for 10 s, followed by 40 cycles of amplification at 95 °C for 10 s and 60 °C for 30 s. Three biological replicates and three technical replicates were performed for all reactions, using *MrACT* as an internal reference gene. Threshold cycle (Ct) values were automatically determined using a qTOWER3G fluorescence quantitative PCR instrument. The relative gene expression was calculated using the 2^−ΔΔCT^ method [71]. The specific primers involved in this study are shown in Supplementary Table S6.

### 4.7. Statistical Analysis

GraphPad Prism 9 was used for data analysis and graphing. The results are expressed as the mean ± standard deviation (SD) from at least three replicate experiments. A one-way ANOVA, followed by Tukey’s test, was used to determine whether there was a statistically significant difference in the different independent groups.

## 5. Conclusions

In this study, seven ACS genes (*MrACS1-7*) were identified in *Myrica rubra* and were characterized by conserved structural features, including the PLN02450 domain (except *MrACS3*), hydrophilic properties, and α/β-rich tertiary structures. Phylogenetic analysis grouped these genes into three clades, revealing evolutionary conservation with Rosaceae species (*Prunus persica* and *Malus domestica*). Segmental duplication events (*MrACS4*-*MrACS5* and *MrACS6*-*MrACS7*) and low Ka/Ks ratios (0.09–0.16) suggest that the purifying selection process has contributed to the maintenance of functional stability. Tissue-specific expression profiling highlighted *MrACS5* as a fruit-specific gene that is significantly upregulated during late developmental stages (57–113 DAP), suggesting its critical role in ethylene-mediated ripening. In contrast, *MrACS7* exhibited broad expression across tissues, implying a regulatory function in basal ethylene metabolism. These findings establish a foundation for deciphering ethylene biosynthesis in *Myrica rubra* and prioritizing *MrACS5* as a key candidate for further functional studies aimed at improving postharvest fruit quality.

## Figures and Tables

**Table 1:** The characteristics of 7 ACSs in *Myrica rubra*.

Gene Name	Number of Amino Acid	Molecular Weight (kDa)	Theoretical pI	Instability Index	Aliphatic Index	Grand Average of Hydropathicity	Subcellular Localization
*MrACS1*	470	52.77	8.22	45.56	83.21	−0.269	Cytoplasm
*MrACS2*	477	53.71	5.84	47.28	82.6	−0.24	Nucleus
*MrACS3*	446	50.18	5.79	50.83	83.77	−0.336	Cytoplasm
*MrACS4*	486	54.60	6.6	43.83	82.84	−0.18	Cytoplasm
*MrACS5*	479	53.64	6.21	50.03	83.63	−0.209	Cytoplasm
*MrACS6*	528	58.41	8.4	50.54	89.6	−0.084	Nucleus
*MrACS7*	551	60.34	8.33	48.73	89	−0.07	Cytoplasm

**Table 2:** Ka/Ks of *MrACS*.

		Ka	Ks	Ka/Ks
*MrACS4*	*MrACS5*	0.17033069606252893	1.9638562679829152	0.08673277104819707
*MrACS6*	*MrACS7*	0.23165164492644064	1.4133469890885786	0.16390288210528203

## Data Availability

The data presented in this study are available in the article and Supplementary Materials.

## Supplementary Materials

The following supporting information can be downloaded at: https://www.mdpi.com/article/10.3390/ijms26104580/s1.
